# Clinical-epidemiological profile of right and left colorectal cancer: exploratory study at the university hospital of USP (2012-2018)

**DOI:** 10.3389/fsurg.2026.1809373

**Published:** 2026-07-20

**Authors:** Aline Moreira Morais, Paulo S. M. Alcântara, Joyce de Cassia Rosa de Jesus, Afonso M. Andrade, Marilia Seelaender, Jose Pinhata Otoch

**Affiliations:** 1 University Hospital of the University of São Paulo (HU-USP), São Paulo, Brazil; 2 CoMeta-HU, University of São Paulo, São Paulo, Brazil; 3 Laboratory for Analysis and Dissemination of Knowledge in Education (LADICE-FEUSP), Faculty of Education, University of São Paulo, São Paulo, Brazil; 4 Department of Surgery, Laboratory of Medical Investigation 26 (LIM26), Hospital das Clínicas, Faculdade de Medicina, Universidade de São Paulo (HC-FMUSP), São Paulo, Brazil; 5 C2PO-USP, University of São Paulo, São Paulo, Brazil

**Keywords:** biomarkers, cachexia, colorectal cancer, inflammation, prognosis

## Abstract

**Introduction:**

Cachexia is a multifactorial syndrome associated with systemic inflammation, nutritional deterioration, and poor prognosis in cancer patients. Colorectal cancer is one of the most prevalent malignancies worldwide, and differences between right- and left-sided tumors may influence clinical outcomes.

**Methods:**

This retrospective observational cohort study included 69 patients who underwent surgical treatment for colorectal cancer at the University Hospital of the University of Sao Paulo between 2012 and 2018. Patients were classified as cachectic (CC; n = 40) or non-cachectic (WSC; n = 29). Clinical, epidemiological, inflammatory, nutritional, and laboratory variables were compared according to cachexia status, primary tumor location, and survival status.

**Results:**

Cachectic patients presented significantly lower BMI, hemoglobin, and albumin levels, as well as higher CRP concentrations, CRP/albumin ratio, GPS, and mGPS values compared with non-cachectic patients. Cachexia was more frequent among patients with right-sided tumors than among those with left-sided tumors, although this difference did not reach statistical significance. Patients who died during follow-up were significantly older than survivors.

**Discussion:**

Cachectic patients with colorectal cancer showed a distinct inflammatory and nutritional profile, supporting the clinical relevance of accessible biomarkers such as CRP, albumin, GPS, and mGPS for patient assessment and risk stratification. Further multicenter studies with larger samples and complete longitudinal follow-up are needed to clarify the prognostic implications of these findings.

## 1 Introduction

Cancer-associated cachexia (CAC) is a severe, multifactorial syndrome characterized by involuntary weight loss and muscle wasting. It is one of the primary contributors to cancer morbidity and mortality and is driven by chronic systemic inflammation (1).

Cachexia is a challenging multifactorial and multiorgan clinical entity, associated with unfavorable outcomes in cancer patients and characterized by pronounced involuntary weight loss, systemic inflammation, metabolic disturbances, and anorexia (2).

The consensus diagnostic criterion for cachexia is a weight loss greater than 5% or a weight loss greater than 2% in individuals already presenting with depletion according to current body weight and height [body mass index (BMI) < 20 kg/m^2^] or skeletal muscle depletion (sarcopenia) (3).

Patients with gastrointestinal cancers, including pancreatic, gastric, and colorectal cancer, are commonly affected by cachexia (4).

Colorectal cancer (CRC) ranks as the third most prevalent neoplasm and the second most common cause of cancer-related deaths worldwide (5).

CRC is a neoplasm affecting the large intestine (colon, rectum, and anus), epidemiologically identified using the International Statistical Classification of Diseases and Related Health Problems, 10th Revision (ICD-10), and subdivided into colon (C18), rectosigmoid junction (C19), rectum (C20), and anus (C21) (6, 7).

In Brazil, CRC is the third most common cancer in men and the second most common in women, with an estimated 45,000 new cases during the period 2023–2025 (7).

Several studies have associated primary tumor location (PTL) with prognosis and survival outcomes. Clinical, histological, and molecular differences between right-sided CRC and left-sided CRC (LCC) have gained increasing attention (6).

The right colon originates from the midgut, while the left colon derives from the hindgut. The different microenvironments of the right and left colon can result in distinct mutation profiles during carcinogenesis (8).

There is substantial evidence suggesting that right-sided CRC and left-sided CRC differ in terms of histological and clinical characteristics, including tumor progression and metastatic potential (9).

Numerous articles have investigated the relationship between primary tumor location and CRC prognosis (5).

Right-sided CRC occurs proximal to the splenic flexure and is considered a tumor of the cecum and ascending colon. Histologically, right-sided tumors often present as mucinous adenocarcinomas with a flat morphology and less frequently as sessile serrated adenomas, making distinction from normal colonic tissue difficult. The luminal contents and fluids in the right colon may delay the appearance of symptoms, contributing to diagnostic delay and a worse prognosis due to advanced tumor stage (10).

Left-sided CRC is defined by tumors occurring at or distal to the splenic flexure, including the descending colon, sigmoid colon, and rectum. Its macroscopic morphology is often polypoid and projects into the intestinal lumen. These tumors frequently cause pain and rectal bleeding, allowing easier detection at earlier stages (11).

In Brazil, despite the high incidence of colorectal cancer, organized population-based screening for asymptomatic individuals remains limited within the public healthcare system. Previous Brazilian studies have reported structural and implementation barriers to colorectal cancer screening, which may contribute to delayed diagnosis, advanced disease at presentation, and reduced survival after diagnosis (12–14).

Therefore, this study aimed to compare clinical, epidemiological, inflammatory and laboratory characteristics between patients with right- and left-sided colorectal cancer, considering the presence of cachexia, in order to explore potential associations with clinical outcomes.

## 2 Methods

### 2.1 Study design and population

**Figure 1:**
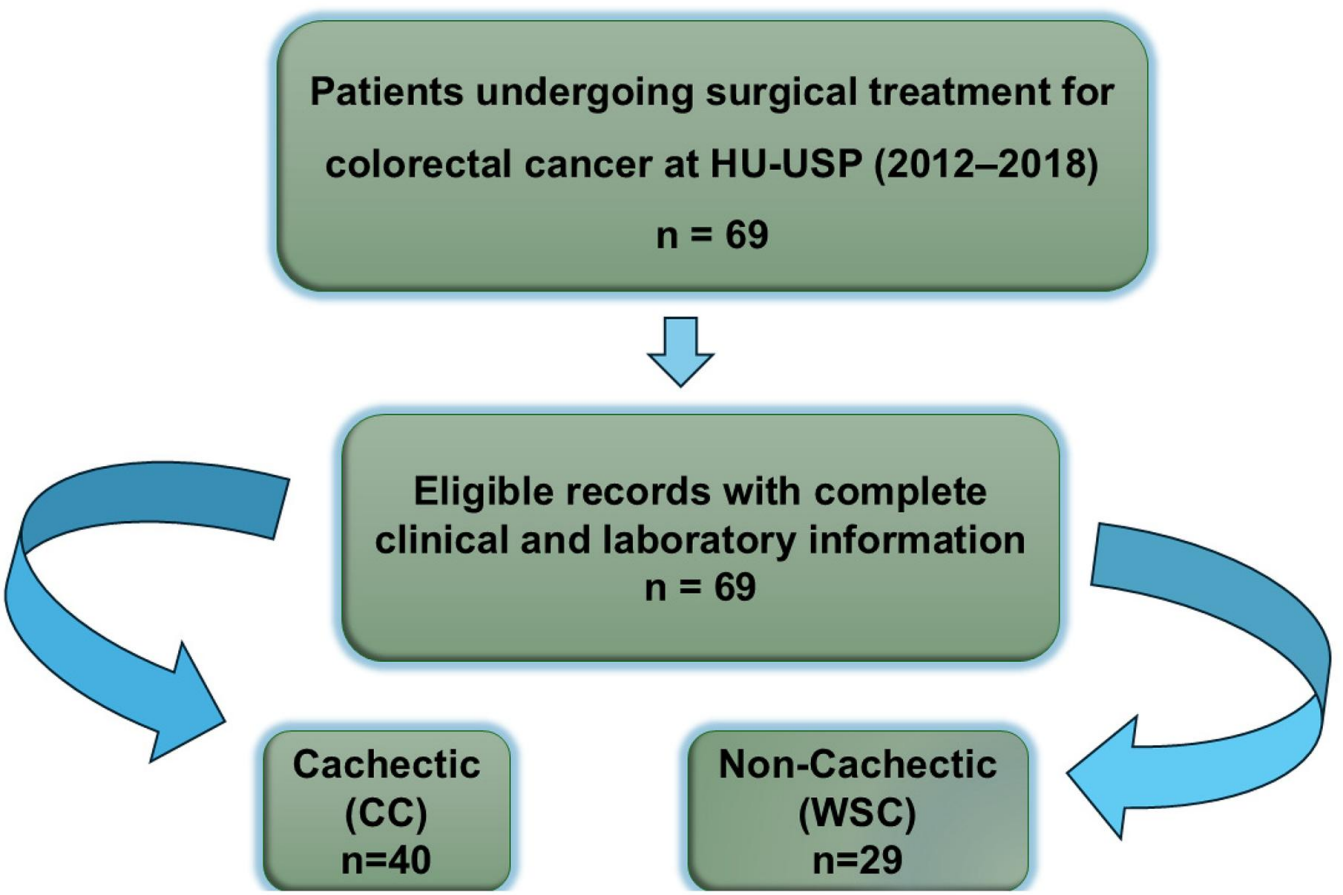
Flowchart of the study population. Patients undergoing surgical treatment for colorectal cancer at HU-USP between 2012 and 2018 were classified according to cachexia status into cachectic (CC) and non-cachectic (WSC) groups.

This observational, cross-sectional, retrospective study was approved by the Research Ethics Committee of the University Hospital of the University of São Paulo (HU-USP), São Paulo, Brazil (CEP-HU/USP No. 2079/23; CAAE 70838623.3.3001.0076). The study population comprised patients who underwent surgical treatment for colorectal cancer at HU-USP between 2012 and 2018. Complete clinical, anthropometric, laboratory, and follow-up data were obtained from medical records. A total of 69 patients met the inclusion criteria and were included in the final analysis (Figure 1).

### 2.2 Cachexia classification

Patients were defined according to the international consensus proposed by Fearon et al. (3). Patients were classified as cachectic when presenting involuntary weight loss greater than 5% during the previous six months, or weight loss greater than 2% in individuals with a body mass index (BMI) lower than 20 kg/m^2^, or in the presence of sarcopenia. According to these criteria, patients were categorized as cachectic (CC; *n* = 40) or non-cachectic (WSC; *n* = 29).

### 2.3 Clinical and laboratory variables

Demographic, clinical, and laboratory variables were extracted from medical records, including age, sex, body mass index (BMI), tumor location, survival status, serum albumin concentration, C-reactive protein (CRP) concentration, Glasgow Prognostic Score (GPS), modified Glasgow Prognostic Score (mGPS), and quality-of-life data obtained through the EORTC QLQ-C30 questionnaire. The selected variables were chosen based on their established association with nutritional status, systemic inflammation, prognosis, and survival in patients with colorectal cancer.

### 2.4 Tumor location and survival assessment

Tumors were classified according to anatomical location as right-sided colon cancer (CD) or left-sided colon cancer (CE). Survival status was assessed using follow-up information available in medical records and categorized as alive or deceased at the end of the observation period.

### 2.5 Statistical analysis

Continuous variables were expressed as mean and standard deviation (SD), whereas categorical variables were presented as absolute frequencies and percentages. Normality was assessed using the Shapiro–Wilk test before group comparisons. Comparisons between groups were performed using Student's t-test for normally distributed variables and the Mann–Whitney U test for non-normally distributed variables. Categorical variables were compared using Pearson's chi-square test or Fisher's exact test when expected frequencies were lower than five. Statistical significance was defined as *p* < 0.05. Statistical analyses and graphical visualizations were performed using R statistical software.

### 2.6 Ethical considerations

All participants provided written informed consent prior to questionnaire administration and data collection. The study was conducted in accordance with the ethical principles established in the Declaration of Helsinki and approved by the Research Ethics Committee of HU-USP.

## 3 Results

Table 1 summarizes the demographic and laboratory characteristics of the study population. The study included 69 patients, of whom 40 (58.0%) were classified as cachectic (CC) and 29 (42.0%) as non-cachectic (WSC). No significant differences were observed between groups regarding age or sex distribution. However, cachectic patients presented significantly lower BMI (23.3 vs. 26.8 kg/m^2^, *p* = 0.006), lower hemoglobin levels (11.3 vs. 13.3 g/dL, *p* < 0.0001), and lower serum albumin concentrations (3.4 vs. 3.9 g/dL, *p* = 0.048). In contrast, CRP levels were significantly higher in the CC group compared with the WSC group (9.0 vs. 6.5 mg/L, *p* = 0.031). These differences are illustrated in Figure 3.

**Table 1:** Sample characteristic. Baseline demographic and laboratory characteristics of the study population. Continuous variables are presented as mean (SD), and categorical variables as frequency and percentage. Comparisons were performed between cachectic (CC) and non-cachectic (WSC) patients.

Variable	Total (*n* = 69)	CC (*n* = 40)	WSC (*n* = 29)	*p*-value
Age (years)	63.2 (14.0)	65.3 (13.8)	60.2 (14.0)	0.134
Female, *n* (%)	33 (47.8)	16 (40.0)	17 (58.6)	0.149
Male, *n* (%)	36 (52.2)	24 (60.0)	12 (41.4)	-
BMI (Kg/m^2^)	24.8 (5.3)	23.3 (5.1)	26.8 (5.0)	0.006
Hemoglobin (g/dL)	12.1 (2.5)	11.3 (2.2)	13.3 (2.4)	<0.0001
CRP (mg/L)	8.0 (4.8)	9.0 (4.8)	6.5 (4.6)	0.031
Albumin (g/dL)	3.6 (0.9)	3.4 (1.0)	3.9 (0.8)	0.048

No significant differences were observed between right-sided and left-sided colorectal cancer regarding age, sex distribution, CRP concentrations, or albumin levels. Cachexia was more frequently observed among patients with right-sided tumors (70.8%) than among those with left-sided tumors (51.1%), although this difference did not reach statistical significance (*p* = 0.185).

**Figure 2:**
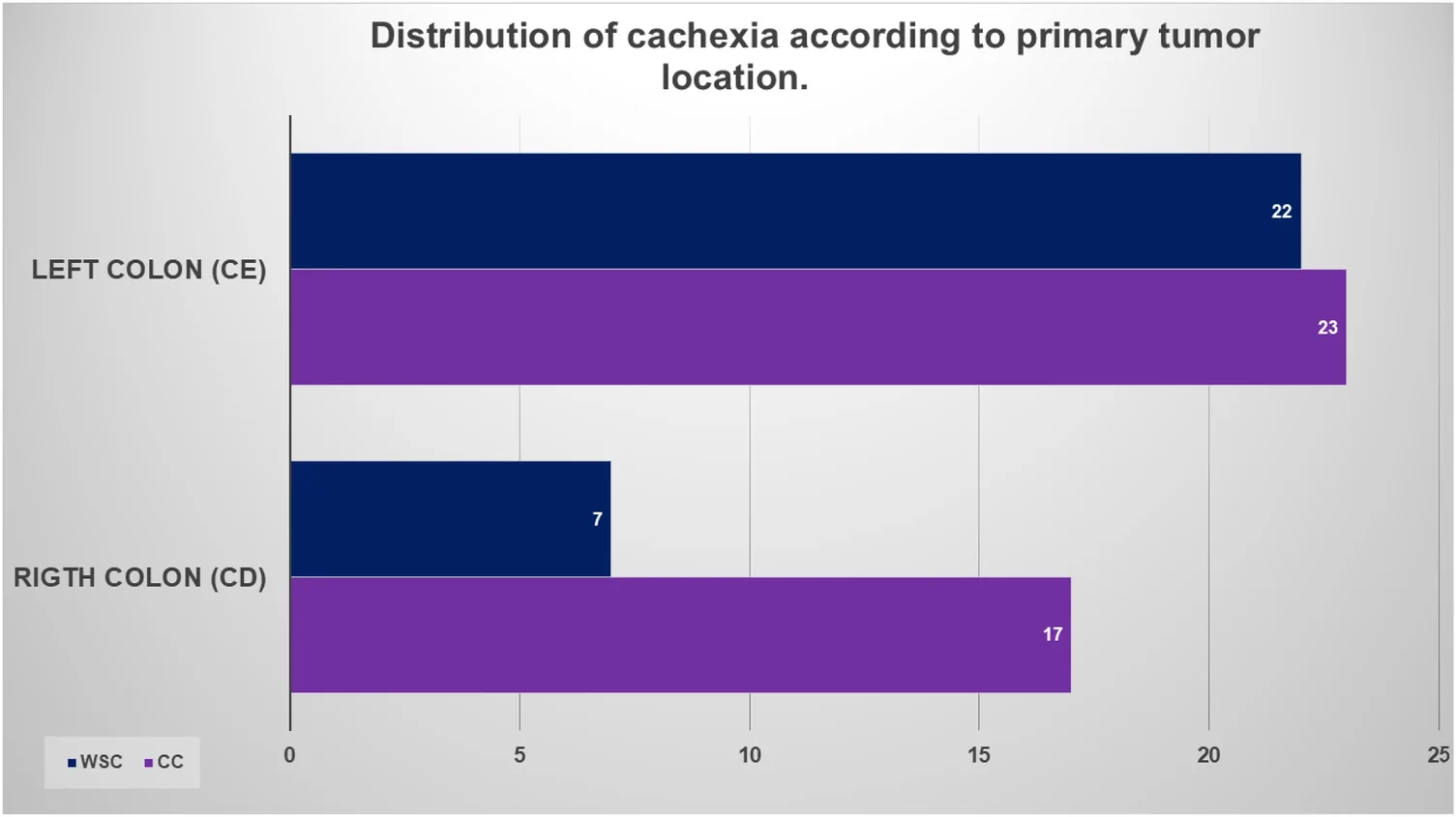
Distribution of cachectic (CC) and non-cachectic (WSC) patients according to primary tumor location. Right-sided tumors were more frequently associated with cachexia (70.8%) than left-sided tumors (51.1%), although the difference did not reach statistical significance (*p* = 0.185).

No significant differences were observed between patients with right-sided and left-sided colorectal cancer regarding age, sex distribution, CRP concentrations, or albumin levels (Table 2). However, cachexia was more frequently observed among patients with right-sided tumors than among those with left-sided tumors (70.8% vs. 51.1%), although this difference did not reach statistical significance (*p* = 0.185). This distribution is illustrated in Figure 2, which shows the absolute number of cachectic and non-cachectic patients according to primary tumor location.

**Table 2:** Right vs. Left Colon. Clinical and laboratory characteristics according to primary tumor location. Comparisons were performed between patients with right-sided colon cancer (CD) and left-sided colon cancer (CE). Continuous variables are presented as mean values and categorical variables as frequency and percentage.

Variable	Right Colon (CD) *n* = 24	Left Colon (CE) *n* = 45	*p*-value
AGE (years)	60.7	64.5	0.328
Female, *n* (%)	12 (50.0%)	21 (46.7%)	0.991
Male, *n* (%)	12 (50.0%)	24 (53.3)	-
CC, *n* (%)	17 (70.8%)	23 (51.1%)	0.185
WSC, *n* (%)	7 (29.2)	22 (48.9)	-
CRP (mg/L)	8.80	7.54	0.313
Albumin (g/dL)	3.50	3.66	0.513

**Figure 3:**
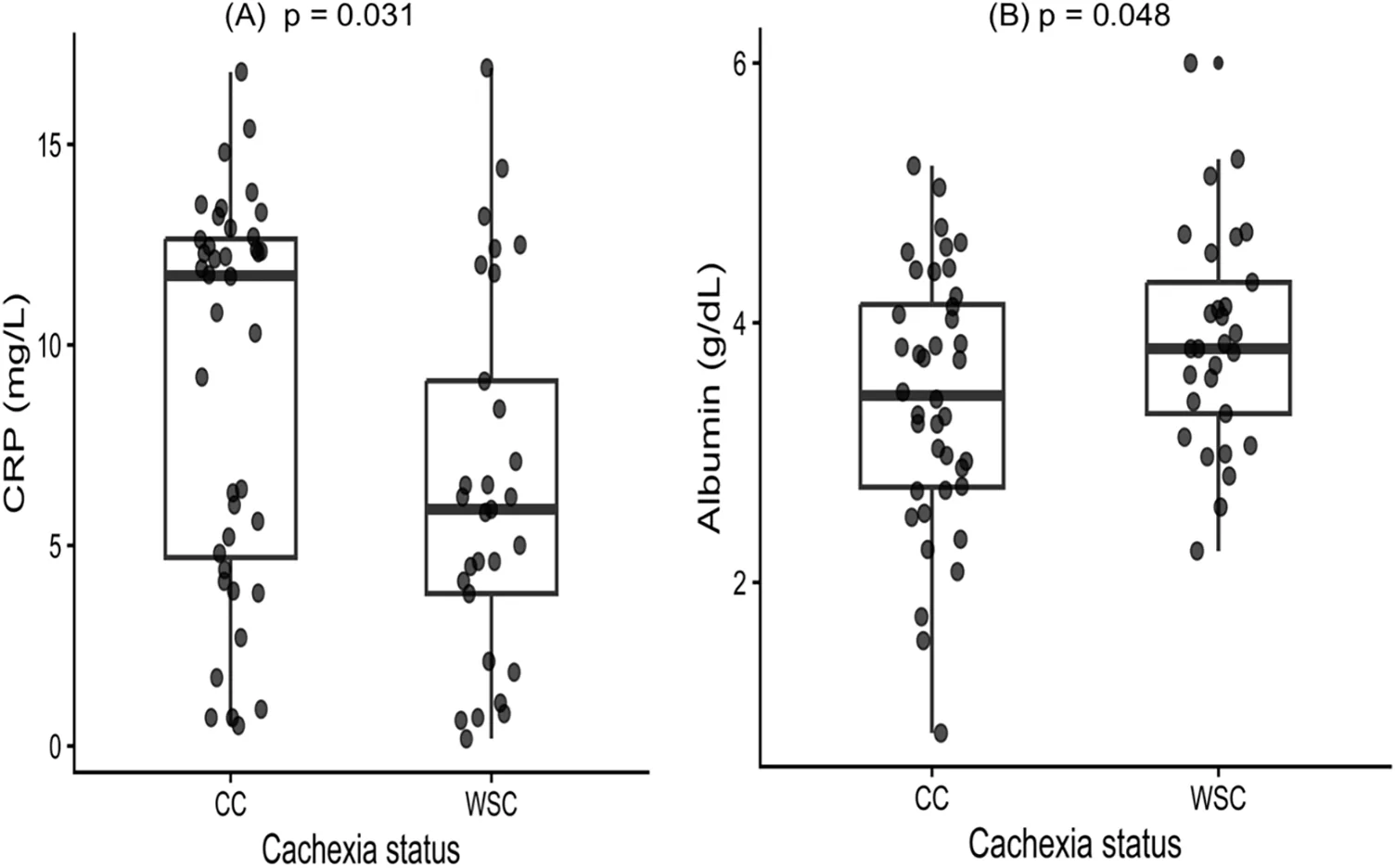
Inflammatory and nutritional biomarkers according to cachexia status. **(A)** Distribution of serum C-reactive protein (CRP) concentrations. **(B)** Distribution of serum albumin concentrations. Cachectic patients (CC) presented significantly higher CRP levels and lower albumin concentrations than non-cachectic patients (WSC). Boxes represent the interquartile range, horizontal lines indicate the median, and whiskers represent data dispersion.

Patients who died were significantly older than survivors (68.7 vs. 61.0 years, *p* = 0.041) (Table 3). No statistically significant differences were observed regarding sex distribution, cachexia status, BMI, hemoglobin, CRP, or albumin levels.

**Table 3:** Mortality according to clinical and laboratory characteristics. Clinical and laboratory characteristics according to survival status. Continuous variables are presented as mean values and categorical variables as frequency and percentage. Comparisons were performed between deceased and surviving patients during the follow-up period.

Variable	Death (*n* = 19)	Alive (*n* = 50)	*p*-value
Age (years)	68.7	61.0	0.041
Female, *n* (%)	6 (31.6)	27 (54.0)	0.013
Male, *n* (%)	13 (68.4)	23 (46.0)	-
CC, *n* (%)	14 (73.7)	26 (52.0)	0.171
WSC, *n* (%)	5 (26.3)	24 (48.0)	-
BMI (kg/m^2^)	24.94	24.75	0.895
Hemoglobin (g/dL)	12.58	11.97	0.364
CRP (mg/L)	8.31	7.85	0.728
Albumin (g/dL)	3.66	3.58	0.759

Cachectic patients presented significantly lower BMI, hemoglobin and albumin levels, as well as higher CRP concentrations, CRP/albumin ratio, GPS and mGPS scores when compared with non-cachectic patients (Table 4).

**Table 4:** Clinical and inflammatory characteristics according to cachexia status. Inflammatory and nutritional characteristics according to cachexia status. Cachectic patients (CC) were compared with non-cachectic patients (WSC). CRP/albumin ratio, Glasgow Prognostic Score (GPS), and modified Glasgow Prognostic Score (mGPS) were evaluated as markers of systemic inflammation and nutritional impairment.

Variable	CC (*n* = 40)	WSC (*n* = 29)	*p*-value
BMI (kg/m^2^)	23.3	26.8	0.006
Hemoglobin (g/dL)	11.3	13.3	<0.001
CRP (mg/L)	9.0	6.5	0.031
Albumin (g/dL)	3.4	3.9	0.048
CRP/Albumin ratio	3.3	1.8	0.012
GPS	1.1	0.5	0.007
mGPS	0.9	0.3	0.003

## 4 Discussion

The present study investigated clinical, inflammatory, and nutritional characteristics of patients with colorectal cancer according to cachexia status and primary tumor location. The main findings were that cachectic patients exhibited significantly lower BMI, hemoglobin, and albumin levels, as well as higher CRP concentrations and worse inflammatory scores (GPS and mGPS). In contrast, no significant differences were observed between right-sided and left-sided colorectal cancer regarding demographic or laboratory characteristics.

The reductions in BMI, hemoglobin, and albumin observed among cachectic patients, together with elevated CRP concentrations, are consistent with the current understanding of cancer cachexia as a multifactorial syndrome characterized by systemic inflammation, progressive nutritional deterioration, and metabolic dysregulation. According to the international consensus proposed by Fearon et al., cancer cachexia results from a complex interaction between reduced nutritional intake and tumor-induced inflammatory responses, leading to ongoing loss of skeletal muscle mass and functional decline. The lower BMI values observed in cachectic patients in the present study reflect this progressive wasting process and support the adequacy of the classification criteria used (3, 15).

Systemic inflammation plays a central role in the pathophysiology of cancer cachexia. Elevated CRP concentrations observed among cachectic patients indicate activation of the acute-phase inflammatory response and may reflect the inflammatory component of nutritional deterioration. Recent evidence has demonstrated that inflammatory biomarkers, particularly CRP and albumin-based indices, are associated with disease progression, treatment response, postoperative complications, and survival outcomes in patients with colorectal cancer and other gastrointestinal malignancies. The significantly higher CRP levels identified in the present study therefore support the concept that systemic inflammation represents a major biological component of cancer cachexia (16–18).

Similarly, serum albumin has been widely recognized as an indicator of both nutritional status and inflammatory burden. Hypoalbuminemia may result from reduced protein synthesis, increased catabolism, and persistent inflammatory activity, all of which are common features of cachexia. In the present study, cachectic patients presented significantly lower albumin concentrations than non-cachectic individuals, reinforcing the close relationship between nutritional deterioration and inflammatory activation. Recent studies have highlighted the prognostic value of albumin and CRP-based biomarkers in colorectal cancer, demonstrating their association with postoperative complications, reduced treatment tolerance, and poorer survival outcomes (16, 17).

The significantly higher GPS and mGPS scores observed among cachectic patients further strengthen the inflammatory profile identified in this cohort. Because both scores combine CRP and albumin measurements, they provide an integrated assessment of systemic inflammation and nutritional status. Recent studies have demonstrated that mGPS is associated with oncological outcomes in rectal cancer and may also serve as a prognostic tool in metastatic colorectal cancer. In this context, the higher GPS and mGPS values identified in cachectic patients suggest a more advanced inflammatory and metabolic impairment, supporting the potential utility of these scores as accessible clinical tools for risk stratification and prognostic assessment (19, 20).

The lower hemoglobin concentrations observed among cachectic patients may also reflect the interaction between chronic inflammation, nutritional deficiency, metabolic dysregulation, and cancer-associated anemia. Cancer cachexia is increasingly recognized as a systemic metabolic disorder involving multilevel alterations in energy metabolism, inflammatory signaling, and inter-organ communication, which may contribute to progressive functional decline and impaired physiological reserve. Previous studies have reported that anemia and low hemoglobin levels are associated with worse prognosis in patients with cancer cachexia, potentially influencing functional status, quality of life, treatment response, and survival (21, 22).

An additional finding of the present study was the association between age and mortality. Patients who died during follow-up were significantly older than survivors, whereas no significant differences were observed regarding cachexia status, BMI, hemoglobin, CRP, or albumin concentrations. Age has consistently been recognized as an important prognostic factor in colorectal cancer, reflecting the combined effects of comorbidities, reduced physiological reserve, frailty, and decreased tolerance to surgical and oncological treatments. Although inflammatory and nutritional biomarkers were associated with cachexia in the present study, age emerged as the only variable significantly associated with mortality, suggesting that patient-related factors may play a particularly important role in long-term outcomes (23).

Although right-sided and left-sided colorectal cancers are known to differ in embryological origin, molecular profile, and tumor biology, no significant differences were observed between the groups in the present study. Recent investigations have reported that right-sided tumors are more frequently associated with older age, female sex, microsatellite instability, and distinct biological behavior. However, these biological differences do not necessarily translate into measurable differences in clinical or laboratory parameters, particularly in smaller cohorts. The absence of significant differences in our study may therefore reflect the limited sample size and predominance of inflammatory and nutritional factors associated with cachexia rather than tumor location itself. Nevertheless, a higher proportion of cachectic patients was observed among individuals with right-sided tumors (70.8% vs. 51.1%), suggesting a potential trend that deserves further investigation in larger cohorts (24, 25).

The present study has some limitations that should be acknowledged. First, its retrospective and cross-sectional design limits causal inference. Second, the relatively small sample size may have reduced the statistical power to detect differences between tumor locations. Finally, the study was conducted at a single institution, which may limit the generalizability of the findings. Nevertheless, important strengths include the availability of detailed clinical, laboratory, inflammatory, nutritional, and quality-of-life data, allowing a comprehensive assessment of cachexia-related characteristics in patients with colorectal cancer.

In conclusion, cachectic patients with colorectal cancer exhibited a distinct inflammatory and nutritional profile characterized by lower BMI, hemoglobin, and albumin levels, as well as higher CRP concentrations and inflammatory scores. These findings reinforce the central role of systemic inflammation in cancer cachexia and support the clinical utility of CRP, albumin, GPS, and mGPS as accessible biomarkers for patient assessment and risk stratification.

### 4.1 Study limitations

This study has some limitations that should be acknowledged. First, the analysis was conducted in a single university hospital, which may limit the generalizability of the findings to other populations and healthcare settings. Second, the retrospective and cross-sectional design limits causal inference and does not allow the establishment of temporal relationships between cachexia, inflammatory biomarkers, tumor location, and clinical outcomes. Third, the relatively small sample size may have reduced the statistical power to detect differences between right-sided and left-sided colorectal cancer groups.

Another important limitation is the possible incompleteness of medical records, particularly regarding long-term follow-up information. For this reason, a formal survival analysis, such as Kaplan–Meier curves or Cox regression, was not performed, as censoring data were not consistently available for all patients. Therefore, mortality was analyzed only as a confirmed clinical status during follow-up. In addition, the absence of genomic and molecular data prevented a more detailed correlation with molecular subtypes of colorectal cancer, such as microsatellite instability or other tumor-specific biological features.

Despite these limitations, the study presents relevant strengths, including the availability of clinical, anthropometric, laboratory, inflammatory, nutritional, and quality-of-life data. This allowed an integrated evaluation of cachexia-related characteristics in patients with colorectal cancer and supported the identification of a distinct inflammatory and nutritional profile among cachectic patients.

## 5 Conclusion

Cachectic patients with colorectal cancer exhibited a distinct inflammatory and nutritional profile characterized by lower BMI, hemoglobin, and albumin levels, as well as higher CRP concentrations, CRP/albumin ratio, GPS, and mGPS values. No significant differences were observed between right-sided and left-sided colorectal cancer regarding age, sex distribution, CRP concentrations, or albumin levels, although cachexia was more frequent among patients with right-sided tumors. These findings reinforce the clinical relevance of cachexia as a condition associated with systemic inflammation and nutritional impairment in colorectal cancer patients. Further multicenter studies with larger sample sizes and complete longitudinal follow-up are needed to validate these observations and better clarify their prognostic implications.

## Data Availability

The datasets presented in this article are not readily available because they contain clinical information obtained from medical records and are subject to institutional and ethical restrictions. Requests to access the datasets should be directed to the corresponding author and will be evaluated according to institutional and Research Ethics Committee requirements.
